# Modeling Radiative Efficiency across Fluorinated Molecules: Bridging Chemistry and Climate Policy for Global Warming Potential Estimations

**DOI:** 10.1021/acs.est.5c16501

**Published:** 2026-02-16

**Authors:** Luís P. Viegas, Matilde A. Susano

**Affiliations:** Coimbra Chemistry Centre-Institute of Molecular Sciences (CQC-IMS), Department of Chemistry, 450424 University of Coimbra, Coimbra 3004-535, Portugal

**Keywords:** radiative efficiency, global warming potential, fluorinated compounds, infrared scaling parameters, Kigali Amendment

## Abstract

Accurate assessment of the climate impact of fluorinated compounds is crucial for guiding regulatory decisions and mitigating global warming. We present a novel methodology for calculating the radiative efficiency of diverse fluorinated molecules with minimized error, adaptable to any electronic structure method and basis set. By incorporating full conformer populations and three scaling parameters, we approximate the experimental infrared spectra more effectively, enhancing the reliability of our predictions. The optimization of vibrational frequencies and intensities for a diverse data set of 38 fluorinated compounds enables us to refine radiative efficiency calculations and seamlessly integrate them into our lifetime calculating protocol. We obtain theoretical global warming potential (GWP) values with well-defined error bars, offering a significant improvement over existing computational methods. This enhanced framework provides a powerful tool for assessing the climate effects of fluorinated compounds, aligning with the objectives of the Kigali Amendment to the Montreal Protocol. By delivering robust and reliable GWP estimates, our methodology informs policy decisions on the phasedown of high-GWP hydrofluorocarbons and the search for sustainable alternatives. Our findings contribute to advancing theoretical approaches for quantifying radiative forcing, supporting global efforts to mitigate anthropogenic climate change.

## Graphical abstract

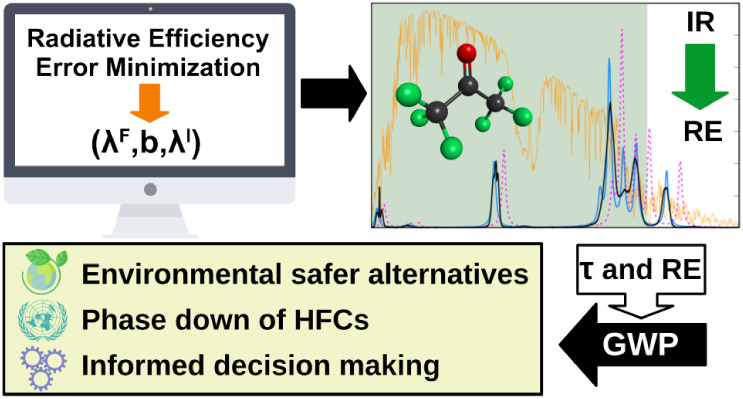

## 1 Introduction

Following the discovery that chlorofluorocarbon (CFC) compounds contribute to ozone depletion and global warming,^1–4^ theoretical and experimental efforts to identify environmentally friendly CFC alternatives have significantly increased. As a result, almost 200 nations adopted the Kigali Amendment to the Montreal Protocol^5^ in October 2016, an international agreement that aims to phase down^6–9^ the production and consumption of hydrofluorocarbons (HFCs), as these second-generation replacements are well-known to have a strong contribution to global warming.^10,11^ Besides dealing with this phasing down process, the Kigali Amendment also promotes alternative technologies by encouraging the adoption of climate-friendly refrigerants and energy-efficient solutions.^12^ For example, hydrofluoroolefins, alcohols, aldehydes, esters, ethers and ketones are potential candidates to replace HFCs in industry.^13^

In the framework of the Kigali Amendment, once a particular compound is categorized as a possible HFC substitute, it becomes crucial to find what is the climate impact of its emission into the atmosphere. The most widely used emission metric in climate policy is the global warming potential (GWP), introduced by the Intergovernmental Panel on Climate Change (IPCC) in 1990,^14^ which compares the heat-trapping ability of different greenhouse gases over a specific time horizon. GWP has been widely used in environmental research^15–22^ and adopted in climate policies: the Kyoto Protocol^4^ adopted GWPs for a time horizon of 100 years, GWP(100), as its metric for implementing a multigas approach. At UNFCCC COP24 ^23^ it was decided to use GWP(100) for reporting national emissions to the Paris Agreement.^24^ The GWP is based on the time-integrated radiative forcing (RF) due to a pulse emission of a unit mass of a gas. It can be given as an absolute GWP for gas *X* (AGWP_*X*_) (usually in W m^–2^ kg^–1^ year) or as a dimensionless value by dividing AGWP_*X*_ by the AGWP of a reference gas, normally CO_2_. Thus, the GWP for gas *X* over a time horizon of *H* years is defined as^25^

1 $${\mathrm{GWP}}_{X}\left(H\right)=\frac{\int_{0}^{H}{\mathrm{RF}}_{X}\left(t\right)dt}{\int_{0}^{H}{\mathrm{RF}}_{{\mathrm{CO}}_{2}}\left(t\right)dt}=\frac{{\mathrm{AGWP}}_{X}\;\left(H\right)}{{\mathrm{AGWP}}_{{\mathrm{CO}}_{2}}\;\left(H\right)}$$

with^26^ ${\mathrm{AGWP}}_{{\mathrm{CO}}_{2}}\left(100\right)=8.947\times 10^{-14}Wm^{-2}year{\left(kgCO_{2}\right)}^{-1}$ . Assuming first order chemical loss for compound *X* and a linear regime consistent with small perturbations in mixing ratio, the radiative forcing of compound *X* can be written^14,25^ as RF_*X*_ = RE_*X*_ · exp­(−*t*/τ_*X*_), where RE_*X*_ is the radiative forcing per unit change in the mixing ratio of compound *X*, a quantity also known as radiative efficiency, RE. The exponential term gives the time-dependent mixing ratio response after the pulse emission, which is then integrated from *t* = 0 to *t* = *H*. The AGWP for gas *X* can then be expressed as^25^

2 $${\mathrm{AGWP}}_{X}\left(H\right)=\frac{10^{9}{\mathrm{RE}}_{X}{\mathrm{M̅}}^{\mathrm{dry atm.}}}{M\left(X\right)m_{\mathrm{tot}}^{\mathrm{dry atm.}}}\tau_{X}\left[1-\exp\left(-\frac{H}{\tau_{X}}\right)\right]$$

where RE_*X*_ is given in W m^–2^ ppbv^–1^ and the 10^9^ factor cancels out the ppbv^–1^ units. Additionally, *M̅* ^dry atm.^ is the average of the molar masses of a dry atmosphere^25^ and $m_{\mathrm{tot}}^{\mathrm{dry atm.}}$ is the total mass of a dry atmosphere.^27^ Both quantities are known with values of *M̅* ^dry atm.^ = 0.02897 kg mol^–1^ and $m_{\mathrm{tot}}^{\mathrm{dry atm.}}=5.1352\times 10^{18}$ kg. For each compound *X*, its AGWP over a time horizon of *H* years is therefore given by eq 2, which depends on three quantities: its *M*(*X*) molar mass (in kg mol^–1^), its atmospheric lifetime (τ_*X*_, in years) and its radiative efficiency (RE_*X*_). While the molar mass is easily determined, obtaining τ_*X*_ and RE_*X*_ is far more challenging, as it requires complex experimental setups or computational methods.

Such complexity is particularly evident in the theoretical calculation of τ_*X*_. Halocarbons in general and fluorocarbons in particular have two main degradation pathways:^25,28–30^ stratospheric photolysis and OH-initiated tropospheric oxidation, such that^25,31^

3 $$\frac{1}{\tau_{X}}\approx \frac{1}{\tau_{X}^{\mathrm{photolysis}}}+\frac{1}{\tau_{X}^{\mathrm{OH}}}$$

where the photolytic process with rate constant *J* depends on the altitude^29,30^ and is only relevant for aldehydes and ketones^28–30^ since these two families of compounds contain a carbonyl group which absorbs radiation in the ultraviolet region due to its *n* → π* transition.^32^ Moreover, the fluorination of aldehydes and ketones results in a bathochromic shift in the UV absorption bands of the carbonyl groups^32–34^ enabling a better overlap with the actinic flux available at lower altitudes and decreasing the value of $\tau_{X}^{\mathrm{photolysis}}$ to a range between a few days and a few weeks.^32–35^ For fluorocarbons possessing hydrogen atoms, other than aldehydes and ketones, photolysis will not be relevant and the atmospheric lifetime will strongly depend on the OH tropospheric oxidation reactions, in such a way we can write

4 $$\tau_{X}\approx \tau_{X}^{\mathrm{OH}}=\frac{1}{k_{OH}\left[OH\right]}$$

where *k*_OH_ is the OH-oxidation rate constant and [OH] is the global average concentration^36^ of OH, usually set at [OH] = 1 × 10^6^ molecules cm^–3^. Consequently, in order to obtain τ_*X*_, the *k*_OH_ oxidation rate constant must be obtained, which can present complicated challenges to theoretical models, particularly due to increasing system size and its associated conformational complexity. To address these issues, we have recently developed^37,38^ and improved^39–44^ a computational protocol for the cost-effective calculation of bimolecular rate constants in the high-pressure limit for the reaction between OH radicals and fluorinated compounds of moderate/large size. This protocol is based on multiconformer transition state theory (MC-TST)^45–51^ and a method for performing transition state sampling called constrained transition state randomization (CTSR).^39,44^ Our MC-TST/CTSR protocol^42^ was very recently tested^42,43^ with 12 different fluorinated compounds belonging to five different families, namely hydrofluoroalcohols, hydrofluoroaldehydes, hydrofluoroesters, hydrofluoroethers and hydrofluoroketones. The calculated rate coefficients at 298.15 K were found to agree, on average, with the recommended experimental values^52^ within a factor of 2 or better.

Having established a cost-effective computational method for calculating $\tau_{X}^{\mathrm{OH}}$ , the remaining component needed to theoretically determine the GWP within the framework of our protocol is the calculation of the RE_*X*_ radiative efficiency. As with the atmospheric lifetime, the RE, which depends on the infrared (IR) absorption cross-section spectrum, can be obtained via experiments or theoretical calculations. Although most Fourier transform IR spectrometer (FTIR) measurements focus on frequencies above ∼500 cm^–1^, the overlooked low-frequency region below this threshold deserves attention, as it represents approximately 16% of the total IR irradiance.^53^ Moreover, obtaining high-quality IR spectra experimentally is challenging due to factors such as impurities, temperature and pressure control, path length limitations, and potential equipment errors—challenges that are especially pronounced for low vapor pressure (less-volatile) substances [ref 54 and references therein]. Consequently, these limitations could hinder future efforts to obtain reliable IR spectra (and thus reliable RE values) for molecules that have not yet been experimentally characterized. Under these constraints, theoretical approaches stand out as the most viable path for accurately determining the RE. The computational investigation of the RE has seen sustained and significant development over the years^6,53,55–64^ with some investigations delving into the framework of machine learning;^65,66^ however, these methods remain in an initial stage of development, and their results are still subject to extensive validation. Although the current methodologies take important conformational aspects into account, they attempt to replicate RE values as accurately as possible using previously published scaling factors for vibrational frequencies that may not be completely adequate to the specific problem being studied—the Kigali Amendment to the Montreal Protocol. In other cases, efforts have gone further, such as in the work by Betowski et al.,^60^ where vibrational intensities were identified as the primary source of error in RE calculations. To address this, scaling factors were also applied to the vibrational intensities, resulting in greater flexibility and improved accuracy (extra parameter). However, in addition to lacking a description of the methodology used to obtain the vibrational intensity scaling factors, many of the molecules included in the database employed in this work would not be permitted or deemed relevant under the frameworks of the Montreal Protocol and/or the Kigali Amendment. This limitation ultimately impacts the reliability of the results for fluorinated compounds of current genuine regulatory and environmental interest.

More recently, Alvarado-Jiménez and Tasinato^64^ have proposed a methodology based on the more accurate anharmonic approach, which in principle should bring the calculated IR spectra closer to the experimental ones and should be considered, when computationally feasible, the recommended methodology. However, such an approach has the well-known drawback of significantly increasing computational costs, which in some cases may act as a deterrent for the routine use of anharmonic calculations. Indeed, this limitation may have influenced the selection of molecules in the study by Alvarado-Jiménez and Tasinato; of the 23 molecules examined, the majority are relatively small, with 20 containing 8 atoms or fewer and only one reaching 12 atoms. Indeed, anharmonic frequency calculations face a 2-fold scaling challenge as molecular size increases. First, the computational cost of an individual frequency calculation rises steeply with the number of atoms. Second, larger molecules possess a more complex conformational landscape, resulting in a greater number of stable conformers that must each be independently evaluated. The total computational effort is therefore compounded: not only does each individual anharmonic frequency calculation become more expensive, but a larger volume of these calculations is required. While there are very useful methodological developments aimed at computationally efficient anharmonic calculations^67–71^ these are currently unavailable in the anharmonic procedures of the open source software we employ.^72^ Therefore, for the molecules studied in this work, a methodology based on scaled harmonic calculations is justified and the only practically applicable choice. All things considered, recent harmonic approaches to RE calculations reflect careful attention to fundamental theoretical aspects, but none are specifically tailored to the specific set of classes of fluorinated compounds of real interest in the context of the Montreal Protocol and its Kigali Amendment. Given these specificities and the current state of development of efficient anharmonic computations, the establishment of an improved computational methodology based on the harmonic approximation is still justified and essential—one that focuses on the enhancement of the accuracy of previously mentioned approaches and ensures reliable theoretical estimates of the RE for the more restricted set of fluorinated molecules of any size in the absence of experimental data.

The objective of this investigation is thus to accurately model the radiative efficiency of any chosen fluorinated compound, in such a way that we can: 1) easily include the RE_*X*_ calculation in our MC-TST/CTSR protocol and 2) adapt and generalize the RE_*X*_ calculation procedure to any model chemistry besides the M08-HX/pcseg-2 ^73,74^ electronic structure calculations used in our MC-TST/CTSR protocol. The procedure presented in this work will allow us to have a fully operating computational protocol for the determination of the GWP of fluorinated molecules, which can be used to make predictions for unstudied systems and assess the discovery of new greener^75,76^ fluorinated molecules with low GWP: providing solutions to reduce human impact on the climate is of utmost importance within the framework of the Kigali Amendment to the Montreal Protocol. At the same time, our procedure will also offer a systematic approach for obtaining precise radiative efficiency values across various methods and basis set combinations, making it accessible and flexible for use by any researcher or research group. Figure 1 outlines our multistep computational process for determining the GWP for fluorinated substances, along with its principal objectives in the framework of the phase-down of HFCs under the Kigali Amendment.

**Figure 1:**
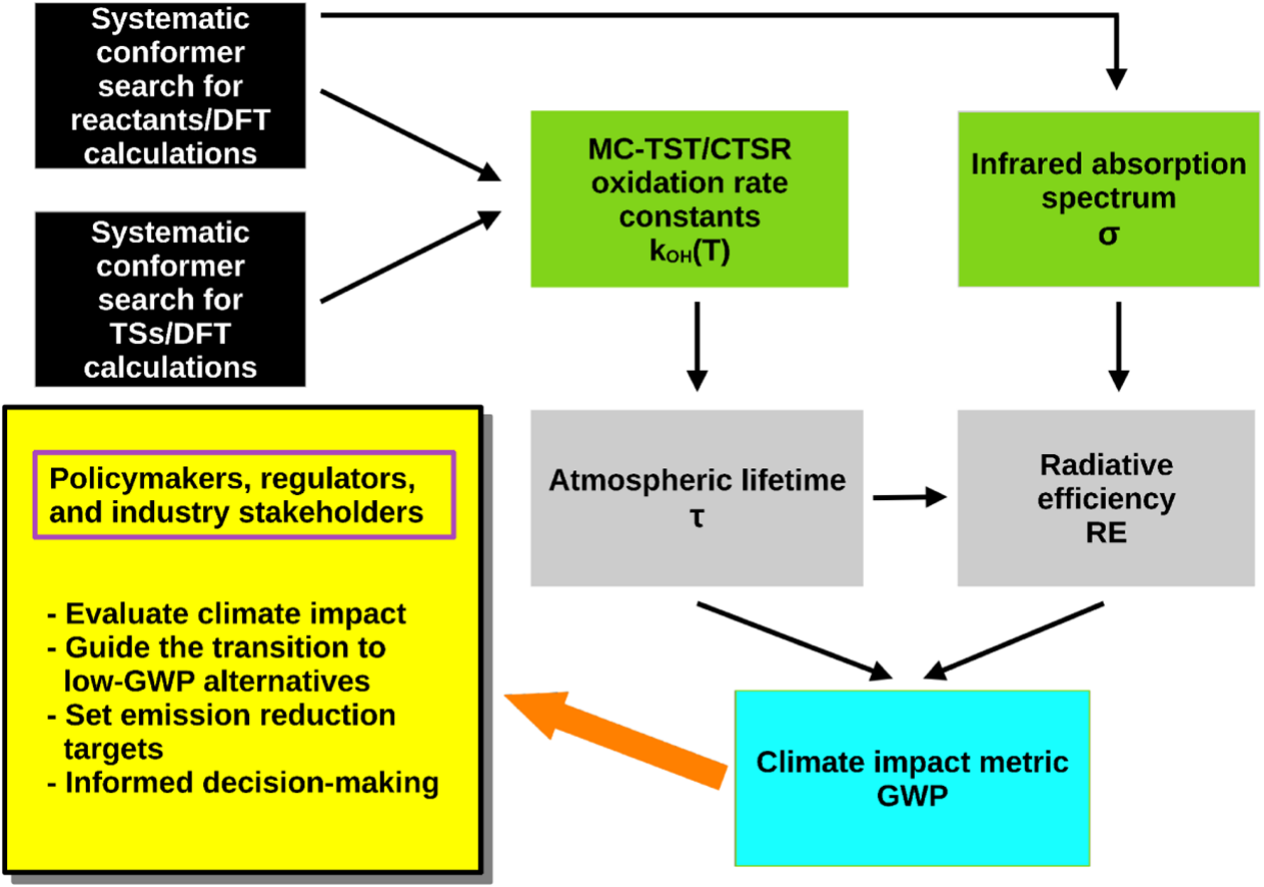
Flowchart outlining the fundamental steps used to theoretically calculate GWP values for fluorinated compounds. GWP predictions can inform policy decisions, emission reduction targets, and the transition to low-GWP alternatives as mandated by the Kigali Amendment to the Montreal Protocol.

In Section 2 we will explain how the RE is calculated and how we can adjust it to any chosen model chemistry, while in Section 3 we apply this procedure and evaluate the values of the RE calculated with the M08-HX/pcseg-2 model chemistry. We finalize with a discussion over the importance of such a tool in the context of the Kigali Amendment.

## 2 Materials and Methods

### 2.1 Quantum Chemistry Calculations

The electronic structure calculations performed in this work exactly reproduce the ones employed in a specific part of our MC-TST/CTSR protocol^39–44^ since that is the methodology we use to calculate the OH oxidation rate constants, which are then employed in the calculation of τ_*X*_. The first step of our MC-TST/CTSR protocol starts with the calculation of the reactant (fluorinated molecule) conformers. This is an important step, since the growing size of these fluorinated compounds will lead to larger molecular chains and an increase in the number of rotatable bonds, therefore resulting in a higher number of rotamers.^77^ An account of these rotamers is essential in obtaining accurate values for the OH oxidation rate constants^38^ and therefore accurate atmospheric lifetimes. The determination of these rotamers starts with confab,^77^ a knowledge-based conformer generation tool that utilizes force fields and a database of allowed torsion angles. We then minimize the generated conformers with the Obminimize program, using the same force field as in the confab calculation, in this case MMFF94.^78^ The confab and Obminimize programs are available in Open Babel.^79^ These structures are then used as starting geometries for optimization at a chosen^80^ level of theory, in this case M08-HX/pcseg-2, which we perform with GAMESS.^72^ We then use a superimposing algorithm^81^ in order to perform a similarity evaluation between all minimized conformers. This identifies identical structures and enantiomers in our pool of optimized reactants, which we can then safely eliminate, leaving us with the final set of *Nconf* unique reactants for any given fluorinated compound *X*.

### 2.2 Calculation of the Radiative Efficiency

The means for computing the RE from computed IR spectra is based on the pioneering work of Pinnock et al.,^82^ who calculated the instantaneous, cloudy-sky, radiative forcing (RF) per unit cross-section as a function of wavenumber for the global and annual mean atmosphere (GAM) and used it to obtain an expression for the final RE as a function of the RF and the absorption cross-section for the compound under study. Papasavva et al.^83^ first demonstrated the use of this method with computed IR spectra using frequencies determined at the MP2/6-31G** level of theory, building on their earlier work validating the use of quantum chemistry methods for the calculation of vibrational frequencies and intensities.^84,85^ This approach has been successful and widely employed in the calculation of the RE for greenhouse gases^6,55–64^ as outlined in the Introduction.

The radiative forcing curve obtained by Pinnock et al.^82^ has been improved over the years. In 2013, Hodnebrog et al.^25^ used the Oslo line-by-line model to generate the radiative forcing at 1 cm^–1^ resolution, an enhancement of the 10 cm^–1^ resolution of Pinnock et al.^82^ More recently, Shine and Myhre^86^ have determined an improved version for the latter spectrally resolved radiative forcing curve^25^ by including a wavenumber dependent stratospheric temperature adjustment (STA) and an improved representation of water vapor and clouds. Note that the STA was originally^25^ included as a 1.1 correction factor for RE_*X*_ in eq 2. As such, the instantaneous RE for compound *X* calculated with the Shine and Myhre RF curve^86^ can be obtained via

5 $${\mathrm{RE}}_{X}^{\mathrm{well‐mixed}}=\overset{3000}{\underset{i=1}{\sum }}F_{i}\left({\mathrm{\nu ̅}}_{i}\right)\sigma_{i}\left({\mathrm{\nu ̅}}_{i}\right)\Delta {\mathrm{\nu ̅}}_{i},in\;Wm^{-2\;}ppbv^{-1}$$

where *F*_*i*_(*ν̅*_*i*_) is the spectrally resolved radiative forcing per unit cross-section, per wavenumber (W m^–2^ cm (cm^2^ molecule^–1^)^−1^) obtained by Shine and Myhre,^86^ evaluated at the waveband *i*, while σ_*i*_(*ν̅*_*i*_) is the absorption cross-section in waveband *i* (cm^2^ molecule^–1^) averaged over the wavenumber increment Δ*ν̅*_*i*_ (cm^–1^), which is 1 cm^–1^ for the Shine and Myhre^86^ curve.

The instantaneous radiative forcing developed by Pinnock et al.^82^ assumes an atmospheric well-mixed distribution of gases across altitude and latitude. However, it has been shown that when such conditions are not met, a multiplicative correction factor for the gas lifetime (τ_*X*_) needs to be applied to the instantaneous (well-mixed) RE calculated in eq 5, which accounts for variables like emission distribution and varying vertical temperature profiles^25^ in order to obtain an effective RE, ${\mathrm{RE}}_{X}^{\mathrm{effective}}$ . The following equations present the gas lifetime corrections, depending on whether the dominant removal process is tropospheric OH degradation (applicable lifetimes ranging from 10^–4^ < τ_*X*_ < 10^4^ years) or photolysis in the stratosphere (10 < τ_*X*_ < 10^4^ years), respectively^25^

6 $$f\left(\tau_{X}\right)=\frac{2.962\tau_{X}^{0.9312}}{1+2.994\tau_{X}^{0.9302}},tropospheric\;OH\;degradation$$

7 $$f\left(\tau_{X}\right)=1-0.1826\tau_{X}^{-0.3339},photolysis\;in\;the\;stratosphere$$

For compounds with τ_*X*_ ≥ 10^4^ years, it has been recommended that no correction should be applied for either case, i.e., *f*(τ_*X*_) = 1.

With the *F*_*i*_(*ν̅*_*i*_) tabulated data available,^86^ the σ_*i*_(*ν̅*_*i*_) absorption cross-section needs to be calculated for any compound *X*. According to IUPAC,^87^ σ_*i*_(*ν̅*_*i*_) (in SI units) is given by

8 $$\sigma_{i}\left({\mathrm{\nu ̅}}_{i}\right)=\frac{\varepsilon_{i}\left({\mathrm{\nu ̅}}_{i}\right)}{N_{A}},in\;m^{2}molecule^{-1}$$

which we will need to express in units of in cm^2^ molecule^–1^. The *ε*_*i*_(*ν̅*_*i*_) variable represents the molar napierian absorption coefficient for band *i* centered on the *k*th vibrational mode which can in turn be calculated as

9 $$\varepsilon_{i}\left({\mathrm{\nu ̅}}_{i}\right)=A_{k}g_{V}\left({\mathrm{\nu ̅}}_{i}-{\mathrm{\nu ̅}}_{k},\gamma_{L},\gamma_{G}\right),in\;cm^{2\;}mol^{-1}$$

where *A*_*k*_ is the integrated absorption coefficient of vibrational mode *k* and *g*_*V*_(*ν̅*_*i*_ - *ν̅*_*k*_, γ_*L*_, γ_*G*_) are the values of the continuous Voigt line shape function evaluated at the specific *ν̅*_*i*_ values. The Voigt function incorporates the combined effects of the Lorentzian broadening resulting from natural line width and pressure (collisional) effects and also of the Doppler broadening (corresponding to a Gaussian profile) resulting from the thermal motion of molecules.^88–90^ In situations where both effects are appreciable, such as in high-resolution (IR or microwave) spectra, the Voigt profile is generally adopted as a more physically realistic representation of the line shape than either a pure Lorentzian or pure Gaussian function alone. While we have adopted the Voigt profile to maintain generality, a simplified treatment utilizing one of the Lorentzian or Gaussian broadening functions mentioned above typically suffices for simulating molecular IR spectra under standard atmospheric conditions. The Voigt line profile is calculated through the convolution of the Gaussian and Lorentzian profiles^89,90^

10 $$g_{V}\left(\mathrm{\nu ̅}-{\mathrm{\nu ̅}}_{k},\gamma_{L},\gamma_{G}\right)=g_{L}*g_{G}=\int_{-\infty }^{+\infty }g_{L}\left(\mathrm{\nu ̅}-{\mathrm{\nu ̅}}^{\prime },\gamma_{L}\right)g_{G}\left({\mathrm{\nu ̅}}^{\prime }-{\mathrm{\nu ̅}}_{k},\gamma_{G}\right)d{\mathrm{\nu ̅}}^{\prime }$$

where γ_*L*_ and γ_*G*_ are the pressure- and temperature-dependent Lorentzian and Gaussian half widths at half-maximum calculated by^88,89,91^

11 $$\gamma_{L}=\gamma_{0}\left(T_{\mathrm{ref}}\right){\left(T_{\mathrm{ref}}/T\right)}^{n}p$$

12 $$\gamma_{G}={\mathrm{\nu ̅}}_{k}\sqrt{\frac{2N_{A}k_{B}T\ln2}{Mc^{2}}}$$

where γ_0_ is the transition dependent collisional broadening parameter at *T*_ref_ (given in cm^–1^ atm^–1^), *n* is a fitting parameter, *p* is the pressure (in atm) and *M* is again the molar mass. While γ_*G*_ is straightforward to evaluate, the same cannot be said about γ_*L*_, since the line-by-line pressure broadening parameters γ_0_ and *n* are very difficult to obtain and are typically unavailable for most molecules.^92^ As a result, we have used γ_*L*_ as a fitting parameter in our procedure, which we will delve into in the next subsection.

The practical evaluation of the Voigt function at each *ν̅*_*i*_ value has been performed with a Fortran90 implementation of a highly efficient novel algorithm^93^ that calculates the Voigt function with an accuracy in the order of 10^–6^.

The *A*_*k*_ integrated absorption coefficient can be calculated theoretically^94^ as

13 $$\begin{array}{ll} A_{k} & =\frac{N_{A}}{12\varepsilon_{0}c^{2}}\left[{\left(\frac{\partial \mu_{a}}{\partial Q_{k}}\right)}^{2}+{\left(\frac{\partial \mu_{b}}{\partial Q_{k}}\right)}^{2}+{\left(\frac{\partial \mu_{c}}{\partial Q_{k}}\right)}^{2}\right]\\ & =\frac{100N_{A}}{12\varepsilon_{0}c^{2}}I_{k},in\;cm\;mol^{-1} \end{array}$$

where eq 1 of ref 94 has to be divided by 4π*ε*_0_ in order to convert to the SI electromagnetic units. *I*_*k*_ represents the transition intensity of the *k*th theoretically calculated vibrational mode, evaluated from the change in dipole moment μ in the molecular principal axes (*a*,*b*,*c*) along the normal mode displacement *Q*_*k*_, which in GAMESS is given in units of (D/Å)^2^ amu^–1^ and needs to be converted to SI units. The quantities *ε*_0_ and *c* represent the vacuum permittivity and the speed of light, respectively, both in SI units.

Substituting eq 9 and 13 in eq 8 one finally obtains the absorption cross-section for band *i* centered on the *k*th vibrational mode

14 $$\sigma_{i}\left({\mathrm{\nu ̅}}_{i}\right)=\frac{100I_{k}}{12\varepsilon_{0}c^{2}}g_{V}\left({\mathrm{\nu ̅}}_{i}-{\mathrm{\nu ̅}}_{k},\gamma_{L},\gamma_{G}\right),in\;cm^{2\;}molecule^{-1}$$

If we consider the possibility that compound *X* has *Nconf* conformers with *N* atoms each, we can rewrite the absorption cross-section by taking into account the 3*N–*6 vibrational modes of each conformer according to

15 $$\sigma_{i}\left({\mathrm{\nu ̅}}_{i}\right)=\overset{Nconf}{\underset{j=1}{\sum }}p_{X_{j}}\overset{3N-6}{\underset{k=1}{\sum }}\frac{100I_{j,k}}{12\varepsilon_{0}c^{2}}g_{V}\left({\mathrm{\nu ̅}}_{i}-{\mathrm{\nu ̅}}_{j,k},\gamma_{L},\gamma_{G}\right)$$

where $p_{X_{j}}$ is the fractional population of conformer *j* calculated via

16 $$p_{X_{j}}=\frac{e^{-(G_{X_{j}}-G_{X_{1}})/k_{B}T}}{\overset{Nconf}{\underset{j=1}{\sum }}e^{-(G_{X_{j}}-G_{X_{1}})/k_{B}T}}$$

with $G_{X_{1}}$ being the lowest Gibbs free energy. Note that the Voigt profile of waveband *i* now depends on the *k*th mode of conformer *j*.

In the spirit of the important developments reported in a previous work,^60^ where vibrational intensities were found to be the main source of error in RE calculations, we will scale both our vibrational modes^57,83^ and respective intensities with the relations ${\mathrm{\nu ̅}}_{j,k}=\lambda^{F}{\mathrm{\nu ̅}}_{j,k}^{\prime }+b$ and $I_{j,k}=\lambda^{I}I_{j,k}^{\prime }$ , where the primed variables are the ones extracted from GAMESS and the (λ^*F*^,*b*,λ^*I*^) set of scaling parameters are targeted for optimization. We can write the final expression for our calculated well-mixed RE value for compound *X* as

17 $${\mathrm{RE}}_{X}^{\mathrm{well‐mixed}}=\overset{3000}{\underset{i=1}{\sum }}F_{i}\left({\mathrm{\nu ̅}}_{i}\right)\overset{Nconf}{\underset{j=1}{\sum }}p_{X_{j}}\overset{3N-6}{\underset{k=1}{\sum }}\frac{100I_{j,k}}{12\varepsilon_{0}c^{2}}g_{V}\left({\mathrm{\nu ̅}}_{i\;}-\;{\mathrm{\nu ̅}}_{j,k},\;\gamma_{L},\;\gamma_{G}\right)\Delta {\mathrm{\nu ̅}}_{i}$$

which we can directly compare to the RE values collected from the most recent World Meteorological Organization scientific assessment on ozone depletion (WMO2022).^26^ We will henceforth designate these data as RE^exp^. A more direct procedure could be envisioned by optimizing the scaling parameters against the errors associated with the calculation of the integrated absorption cross-section given by

18 $$S\left({\mathrm{\nu ̅}}_{1},{\mathrm{\nu ̅}}_{2}\right)=\int_{{\mathrm{\nu ̅}}_{1}}^{{\mathrm{\nu ̅}}_{2}}\sigma \left(\mathrm{\nu ̅}\right)d\mathrm{\nu ̅}\approx \overset{3000}{\underset{i=1}{\sum }}\sigma_{i}\left({\mathrm{\nu ̅}}_{i}\right)\Delta {\mathrm{\nu ̅}}_{i}$$

with the σ_*i*_(*ν̅*_*i*_) cross sections being given by eq 15. However, the calculation^26^ of RE^exp^ is performed by using multiple sources of cross-section data and reanalysis, with these details not being disclosed. Because the actual final form of cross-section data behind each individual RE^exp^ calculation is unknown, obtaining the corresponding integrated absorption cross-section value is not possible. Fortunately, our approach employs the exact same *F*_*i*_(*ν̅*_*i*_) radiative forcing curve^86^ than the one used in the WMO2022 report. This means that minimizing the errors associated with ${\mathrm{RE}}_{X}^{\mathrm{well‐mixed}}$ is equivalent to minimizing the errors associated with the σ_*i*_(*ν̅*_*i*_) cross sections, weighted by the *F*_*i*_(*ν̅*_*i*_) radiative forcing. This weighting function effectively prioritizes the C–F bond stretching region, which is the most critical region of the spectrum for evaluating the RE of fluorinated compounds.

The effective RE, which is the quantity that actually enters the calculation of AGWP_*X*_(*H*) in eq 2, can be then theoretically calculated by

19 $${\mathrm{RE}}_{X}^{\mathrm{effective}}=f\left(\tau_{X}\right){\mathrm{RE}}_{X}^{\mathrm{well‐mixed}}$$

The effective RE values that enter the GWP calculation in the WMO2022 report also include a relatively small low frequency IR absorption adjustment, since the vast majority of experimentally measured IR absorption spectra do not provide data below ∼500 cm^–1^. Our ${\mathrm{RE}}_{X}^{\mathrm{well‐mixed}}$ values given by eq 17 include this low frequency data by definition. In addition, the WMO2022 report includes a tropospheric adjustment to the calculation of ${\mathrm{RE}}_{X}^{\mathrm{well‐mixed}}$ .

### 2.3 Calculation of the Scaling Parameters

In order to calculate the final set of (λ^*F*^, *b*, λ^*I*^) scaling parameters, we developed the procedure presented below. It should be noted that although the scaling parameters we calculated in this work were optimized for the M08-HX/pcseg-2 level of theory, the procedure is valid for any combination of electronic structure method and basis set. This process can be summarized according to the following steps:

1. Define a molecular data set from WMO2022 ^26^ and gather the experimental well-mixed RE values for a number of selected compounds (RE^exp^). In this work, the selection of fluorinated compounds was done by consulting Table A-5 from the WMO’s Annex, with the chosen compounds being marked with the R2 footnote label stating that the “radiative efficiency was taken from the recommendation given in Hodnebrog et al.^95^ ″ with this recommendation being “based on a combination of literature review of experimental data and reanalysis″;
2. Define our (*λ* ^*F*^, *b*, *λ* ^*I*^) grid by specifying the domain over which *λ* ^*F*^, *b* and *λ* ^*I*^ may vary;
3. Perform a conformational analysis (subsection [Sec sec2.1]) for each molecule of our chosen molecular dataset;
4. Selecting a value for *γ*_*L*_, calculate the ${\mathrm{RE}}_{X}^{\mathrm{well‐mixed}}$ values (subsection [Sec sec2.2]) for all grid points defined in step 2, for each compound *X* of the molecular dataset;
5. Calculate the respective mean absolute percentage error (MAPE) for each set of (*λ* ^*F*^, *b*, *λ* ^*I*^) values from the grid;
6. Repeat the two previous steps with different values of *γ*_*L*_. Compare the set of MAPE values associated with each *γ*_*L*_ and select the one yielding the lowest MAPE values;
7. For the predetermined *γ*_*L*_ value, find the approximate location of the MAPE (*λ* ^*F*^, *b*, *λ* ^*I*^) global minimum. This can be accomplished by mapping this function via multidimensional interpolation^96–98^ over a dense grid in the (*λ* ^*F*^, *b*, *λ* ^*I*^) parameter space.
8. Perform a refined minimization of the MAPE by recalculating the ${\mathrm{RE}}_{X}^{\mathrm{well‐mixed}}$ values. This process utilizes a dense grid of points in the parameter space adjacent to the initial, interpolated (*λ* ^*F*^, *b*, *λ* ^*I*^) location determined in the previous step, with the goal of finding a new set of (*λ* ^*F*^, *b*, *λ* ^*I*^) parameters that lower the MAPE value of the previous step.

Considering the core objective of this work—developing a method to derive scaling factors for the accurate determination of radiative efficiencies across a broad range of fluorinated compounds—we constructed our data set using various classes of organic compounds with accurate experimental well-mixed RE values^26^ (RE^exp^). The presence of these different classes allows us to have compounds with a wide range of chemical environments affecting the important C–F bond, which strongly absorbs in the IR region of the electromagnetic spectrum that overlaps with the peak of the spectral radiative forcing per unit absorption cross section^86^ known as the atmospheric window^86,99^ in the region of 700–1300 cm^–1^. It is well established that the unhindered transmission of outgoing longwave radiation via the atmospheric window constitutes a fundamental cooling pathway for the terrestrial system. Consequently, the introduction of molecular species that exhibit absorption cross sections within these transparent spectral frequencies (see Figure 2) imposes a significant radiative imbalance, leading to an intensified greenhouse effect. To evaluate these impacts, the present study utilizes the WMO2022 report^26^ to select a diverse suite of compounds from various classes focusing on those of moderate molecular size (*N̅* ≈ 11 ± 3 and *N*_max_ = 19). The utilization of the WMO2022 report guarantees that the RE^exp^ values were calculated using the most recent radiative forcing curve^86^ and that the GWP values use the most recent values for the atmospheric lifetimes (τ_*X*_).

**Figure 2:**
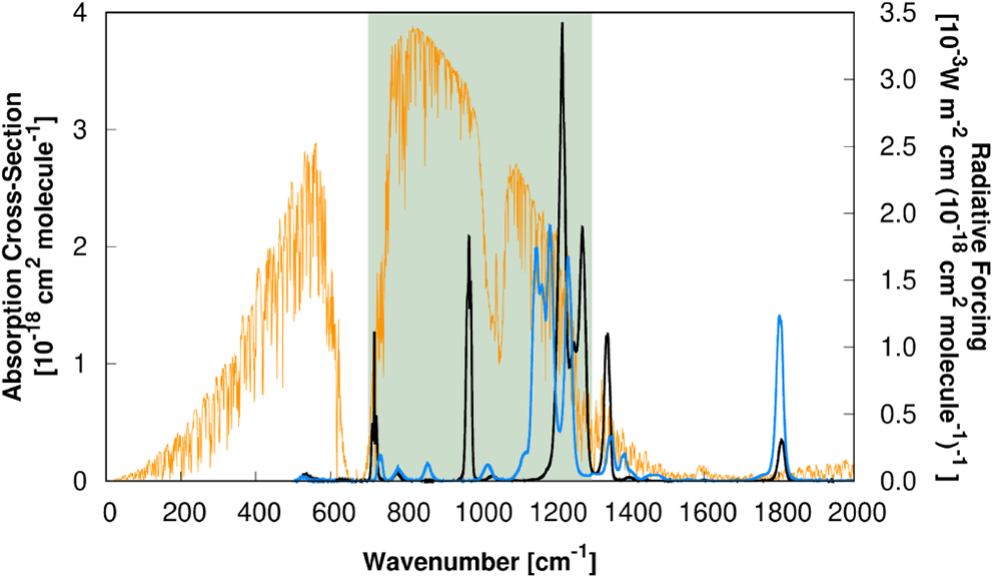
Experimental^100,101^ absorption cross-section spectra [σ*_i_* (*ν̅_i_*)] of ethyl 2,2,2-trifluoroacetate (solid blue line) and 1,1,1,3,3,3-hexafluoropropan-2-one (solid black line) superimposed to the radiative forcing^86^ [*F_i_* (*ν̅_i_*)] per unit cross-section (solid orange line). The light-green transparent rectangle represents the atmospheric window.

The choice of λ^*F*^, *b* and λ^*I*^ domain intervals was made according to what is commonly used in the literature. For λ^*F*^ we chose an interval of [0.90, 1.04] (with steps of 0.02) based on typical published values^102–104^ for vibrational scaling factors. Similarly, for the *b* parameter we chose^57,83^ an interval of [1, 15] cm^–1^ (with steps of 1 cm^–1^). In contrast, information about λ^*I*^ intensity scaling values is much less frequent in the literature.^105^ However, although there is no detailed explanation of how the following values were obtained, Betowski et al.^60^ report λ^*I*^ scaling factors of 0.699 and 0.570 for calculations performed at the B3LYP/6-31G* and B3LYP/6-311++G** levels of theory, respectively. Thus, we opted for an interval of [0.5, 1.0] with steps of 0.1 for the λ^*I*^ scaling factors, which resulted in a (λ^*F*^, *b*, λ^*I*^) grid of 8 × 16 × 6 = 768 points for each fixed value of γ_*L*_.

The MAPE, which is our objective function to be minimized in the procedure above, was calculated according to

20 $$\mathrm{MAPE}=\frac{1}{n}\overset{n}{\underset{i=1}{\sum }}\left|\frac{{\mathrm{RE}}_{i}^{\exp}-{\mathrm{RE}}_{i}^{\mathrm{well‐mixed}}}{{\mathrm{RE}}_{i}^{\exp}}\right|\times 100\%$$

where ${\mathrm{RE}}_{i}^{\mathrm{well‐mixed}}$ is our theoretical prediction for the RE of compound *i* calculated at the M08-HX/pcseg-2 level, ${\mathrm{RE}}_{i}^{\exp}$ is the associated experimental RE prediction extracted from the WMO2022 report and *n* = 38 is the number of compounds in our molecular data set: five hydrofluorocarbons, five perfluorocarbons, five hydrofluoroolefins, five hydrofluoroethers, five hydrofluoroalcohols, three hydrofluoroesters, five hydrofluoroaldehydes and five hydrofluoroketones. We will henceforth refer to this data set as the CFM2025 data set with CFM standing for concise fluorinated molecule.

## 3 Results and Discussion

### 3.1 Radiative Efficiencies in the CFM2025 Data Set

We started the ${\mathrm{RE}}_{X}^{\mathrm{well‐mixed}}$ calculations with γ_*L*_ = 10 cm^–1^. Increasing γ_*L*_ to 12.5 cm^–1^ led to a generic increase of the MAPE values, so we procedeed in dropping the value of γ_*L*_ to 7.5 cm^–1^. After observing a decrease in MAPE values, we continued with this approach and arrived at a value of γ_*L*_ = 4.5 cm^–1^.

In the following step we then used the MAPE values associated with γ_*L*_ = 4.5 cm^–1^ and performed a multidimensional interpolation over a dense grid in the (λ^*F*^, *b*, λ^*I*^) parameter space. The Fortran90 interpolation code^98^ provided four possibilities for interpolating functions, but we tested a few more in order to evaluate the error of the interpolating function against the provided MAPE values at the grid points. This led us to the final choice of the *C* ^4^-continuous Wendland function for three dimensions:^106^ $\psi_{4,2}={\left(1-r\right)}_{+}^{6}\left(35r^{2}+18r+3\right)$ . The interpolation procedure yielded a calculated MAPE value of 5.93% corresponding to the following interpolated scaling parameters: $\lambda_{\mathrm{interp}}^{F}=0.978$ , *b*_interp_ = 1.328 cm^–1^ and $\lambda_{\mathrm{interp}}^{I}=0.861$ . Using these parameters in the actual calculation of ${\mathrm{RE}}_{X}^{\mathrm{well‐mixed}}$ for our CFM2025 data set yields a MAPE of 6.15% and a mean absolute error (MAE) of 0.014 W m^–2^ ppbv^–1^.

In the final step, we recalculated ${\mathrm{RE}}_{X}^{\mathrm{well‐mixed}}$ for our CFM2025 data set in a region around the interpolated scaling parameters. With this procedure we were able to minimize the MAPE to a value of 6.00% with the final scaling parameters being: λ^*F*^ = 0.978, *b* = 0.395 cm^–1^ and λ^*I*^ = 0.855. We should note that the M08-HX/pcseg-2 unscaled calculations (λ^*F*^ = 1, *b* = 0 cm^–1^ and λ^*F*^ = 1) yield a higher error, namely MAPE = 9.82%.

Our detailed results for the CFM2025 data set can be seen in Table 1 where, besides comparing our RE^well‑mixed^ values with the recommended^26^ ones (RE^exp^) we also report: 1) the number of conformers for each fluorinated compound and 2) a comparison between the calculated GWP(100) values based on our RE^well‑mixed^ results and the latest recommended GWP(100) values.^26^ Note that our calculated RE and GWP use the atmospheric lifetimes also reported in WMO2022.^26^

**Table 1:** Comparison between Our RE^well‑mixed^ (In W m^–2^ ppbv^–1^) and GWP(100) Values Obtained for the CFM2025 Dataset, (This Work, Obtained with *λ^F^* = 0.978, b = 0.395 cm^–1^ and *λ^I^* = 0.855) and the Most Recent Recommended Values Published in the WMO2022 report^26^

					RE^well‑mixed^		GWP(100)	
Identifier/name	Formula	CASRN	# conformers	τ (Ref 26)	Ref 26	This work	Ref 26	This work
HFC-23	CHF_3_	75-46-7	1	228 years	0.193	0.196	14700	14160
HFC-134a	CH_2_FCF_3_	811-97-2	1	13.5 years	0.173	0.176	1470	1417
HFC-227ea	CF_3_CHFCF_3_	431-89-0	1	35.8 years	0.278	0.279	3580	3407
HFC-236fa	CF_3_CH_2_CF_3_	690-39-1	1	213 years	0.253	0.268	9120	8818
HFC-365mfc	CH_3_CF_2_CH_2_CF_3_	406-58-6	2	8.86 years	0.240	0.260	969	933
PFC-14	CF_4_	75-73-0	1	50000 years	0.099	0.104	7490	7476
PFC-116	C_2_F_6_	76-16-4	1	10000 years	0.263	0.261	12600	11866
PFC-218	C_3_F_8_	76-19-7	1	2600 years	0.274	0.274	9500	8884
PFC-31-10	n-C_4_F_10_	355-25-9	2	2600 years	0.374	0.374	10200	9600
PFC-41–12	n-C_5_F_12_	678-26-2	4	4100 years	0.412	0.412	9390	8810
HFO-1225ye(E)	(E)-CF_3_CF = CHF	5595-10-8	1	5.7 days	0.259	0.248	<1	0.1
HFO-1234ze(E)	(E)-CF_3_CH = CHF	29188-24-9	1	19 days	0.284	0.281	1	1
HFO-1234yf	CF_3_CF = CH_2_	754-12-1	1	12 days	0.238	0.238	<1	0.5
HFO-1243zf	CF_3_CH = CH_2_	677-21-4	1	9 days	0.177	0.201	<1	0.3
HFO-1261zf	CH_2_FCH = CH_2_	818-92-8	2	0.9 days	0.059	0.064	≪1	0.002
HFE-125	CHF_2_OCF_3_	3822-68-2	2	101.7 years	0.420	0.397	12900	11578
HFE-236ea2 (desflurane)	CHF_2_OCHFCF_3_	57041-67-5	7	13.7 years	0.482	0.431	2530	2133
HFE-236fa	CF_3_CH_2_OCF_3_	20193-67-3	3	7.56 years	0.393	0.381	1300	1019
HFE-245cb2	CF_3_CF_2_OCH_3_	22410-44-2	2	4.99 years	0.365	0.334	754	645
HFE-263mf	CF_3_CH_2_OCH_3_	460-43-5	3	28 days	0.216	0.203	2	2
2,2,2-Trifluoroethanol	CF_3_CH_2_OH	75-89-8	1	163 days	0.202	0.214	30	35
2,2,3,3,3-Pentafluoropropan-1-ol	CF_3_CF_2_CH_2_OH	422-05-9	2	168 days	0.289	0.285	35	32
3,3,3-Trifluoropropan-1-ol	CF_3_CH_2_CH_2_OH	2240-88-2	3	16 days	0.221	0.234	<1	0.8
1,1,1,3,3,3-Hexafluoropropan-2-ol	(CF_3_)_2_CHOH	920-66-1	1	1.88 years	0.334	0.331	219	195
2,2,3,3-Tetrafluoropropan-1-ol	CHF_2_CF_2_CH_2_OH	76-37-9	6	92.4 days	0.257	0.244	16	13
Vinyl 2,2,2-trifluoroacetate	CF_3_C(O)OCH = CH_2_	433-28-3	6	1.7 days	0.261	0.325	≪1	0.01
Ethyl 2,2,2-trifluoroacetate	CF_3_C(O)OCH_2_CH_3_	383-63-1	7	69 days	0.315	0.277	11	9
Allyl 2,2,2-trifluoroacetate	CF_3_C(O)OCH_2_CH = CH_2_	383-67-5	49	1.5 days	0.334	0.306	≪1	0.01
3,3,3-Trifluoropropanal	CF_3_CH_2_CHO	460-40-2	2	2.73 days	0.173	0.168	≪1	0.02
Trifluoroacetaldehyde	CF_3_CHO	75-90-1	1	2.7 days	0.167	0.163	≪1	0.02
2,2,3,3,3-pentafluoropropanal	CF_3_CF_2_CHO	422-06-0	2	1.4 days	0.202	0.226	≪1	0.01
4,4,4-trifluorobutanal	CF_3_CH_2_CH_2_CHO	406-87-1	7	1.8 days	0.163	0.165	≪1	0.01
2,2,3,3,4,4,4-heptafluorobutanal	CF_3_CF_2_CF_2_CHO	375-02-0	8	1.1 days	0.250	0.280	≪1	0.004
1,1,1-Trifluoropropan-2-one	CF_3_C(O)CH_3_	421-50-1	1	16 days	0.205	0.199	3	0.7
1,1,1-Trifluorobutan-2-one	CF_3_C(O)CH_2_CH_3_	381-88-4	5	0.8 days	0.205	0.187	<1	0.002
1-fluoropropan-2-one	CH_3_C(O)CH_2_F	430-51-3	2	16 days	0.046	0.060	<1	0.3
1,1,1,3,3,3-hexafluoropropan-2-one	CF_3_C(O)CF_3_	684-16-2	1	18 days	0.289	0.284	3	3
perfluoro-2-methylpentan-3-one	CF_3_CF_2_C(O)CF(CF_3_)_2_	756-13-8	19	7 days	0.407	0.427	<1	0.5

The agreement between our GWP(100) values and the recommended^26^ ones is excellent, with an associated MAPE value of only 7.8%. However, it only reflects 23 compounds from our CFM2025 data set, since the remaining 15 are reported^26^ as being below 1, see Table 1. Notably, our calculations show that all our GWP(100) calculations for these 15 compounds also yield values below 1. Such a good agreement was expected, since the main difference between both sets of GWP calculations is the value of the RE—the lifetime is the same. However, it is important to note that the GWP(100) values in the WMO2022 report include a tropospheric adjustment in eq 19, which is absent from our calculations. The tropospheric adjustment is generically assumed to be^26^ 0 ± 13%, and for the four molecules in Table 1 for which the value of the experimental and calculated RE^well‑mixed^ is the same (PFC-218, PFC-31-10, PFC-41-12 and HFO-1234yf), we calculated ${\mathrm{RE}}_{X}^{\mathrm{effective}}$ and extracted values of 2.2, 1.6, 1.9 and 2.2% for the tropospheric adjustment, respectively. This methodological difference accounts for the small variations between our calculated GWP(100) values and those in the WMO2022 report when ${\mathrm{RE}}_{i}^{\mathrm{well‐mixed}}$ and ${\mathrm{RE}}_{i}^{\exp}$ are equal. The variety of results for ${\mathrm{RE}}_{i}^{\exp}$ (the highest ${\mathrm{RE}}_{i}^{\exp}$ value is 228 times higher than the lowest ${\mathrm{RE}}_{i}^{\exp}$ value) was the reason why we chose a relative error measure (MAPE) as the objective function to minimize, instead of an absolute error measure (MAE). Clearly, a difference of, for example, 0.003 W m^–2^ ppbv^–1^ between ${\mathrm{RE}}_{i}^{\mathrm{well‐mixed}}$ and ${\mathrm{RE}}_{i}^{\exp}$ carries different significance depending on whether ${\mathrm{RE}}_{i}^{\exp}$ is 0.004 or 0.464 W m^–2^ ppbv^–1^. Usage of the MAPE accounts for this variation, allowing us to accurately reflect it in our results.

To evaluate the robustness of our approach, we have compared our results against previous works that have also considered a fitting of scaling parameters and an evaluation of the quality of the respective RE^well‑mixed^ values. Specifically, we have evaluated the methodology outlined in three publications^53,57,60^ against our CFM2025 data set. Two of these previous publications^57,60^ present a unique challenge: they derived their scaling parameters by fitting their RE^well‑mixed^ values against experimental values different from the ones included in the WMO2022 report and they also calculated RE^well‑mixed^ using an older radiative forcing curve.^82^ Consequently, testing the parameters from these two publications serves as a valuable assessment of their long-term robustness against updated scientific standards. On the other hand, the scaling parameters reported by Van Hoomissen et al.^53^ were calculated by fitting their data to the WMO2022 report and using the most recent radiative forcing curve^86^ to calculate RE^well‑mixed^, as we did with our approach. It should also be mentioned that within this set of four investigations, the one reported in this work is the only one using the more fundamentally sound Voigt line shape profile and the only one that simultaneously fits the three scaling parameters. Bravo et al.,^57^ Betowski et al.^60^ and Van Hoomissen et al.^53^ used a Gaussian line shape function, which we coded for comparison purposes as

21 $$g_{G}\left({\mathrm{\nu ̅}}_{i}-{\mathrm{\nu ̅}}_{j,k},\Gamma \right)=\frac{1}{\Gamma \sqrt{\pi }}\exp\left[-\frac{{\left({\mathrm{\nu ̅}}_{i}-{\mathrm{\nu ̅}}_{j,k}\right)}^{2}}{\Gamma^{2}}\right],in\;cm$$

where Γ is the full width at half-maximum (fwhm). Table 2 shows a summary of the comparison between the four investigations, where we can observe the distinctive features of each implementation.

**Table 2:** Comparison between the Calculated MAPE for the CFM2025 Dataset (eq 20) Using Four Different Theoretical Approaches [This Work, Refs 57,60 and 53 with an Indication of the Level of Theory, Line Shape Profile Details and Scaling Parameters Used in Each One^a^

Level of theory	Line shape/fwhm (cm^–1^)	λ^*F*^	*b* (cm^–1^)	λ^*I*^	MAPE (%)
M08-HX/pcseg-2 [this work]	Voigt/9	0.978	0.395	0.855	6.00
B3LYP/6-31G(d,p)^57^	Gaussian/14	0.977	11.664	1.000	6.39
B3LYP/6-31G(d)^60^	Gaussian/7	0.960	0.000	0.699	16.51
ωB97X-D/def2-TZVPPD^53^	Gaussian/20	0.95655	20.286	1.000	27.82

a Note that the fwhm reported in this work was calculated as fwhm = 2 γ*_L_*.

Table 2 shows that the methodology applied in this work yields the lowest MAPE. However, it should be mentioned that the results obtained with the parameters calculated by Bravo et al.^57^ are also excellent, despite having been obtained with a fit to data concerning only six perfluorocarbons and in the conditions mentioned above. In contrast, the scaling parameters obtained in this work, and even more so in the works of Betowski et al.^60^ and Van Hoomissen et al, were obtained by considering a larger and more diverse data set of chemical substances: 38, 235, and 118 molecules, respectively. We should also note that Betowski et al.^60^ calculated the RE^well‑mixed^ values at several different levels of theory. We chose the B3LYP/6-31G­(d) level of theory from their work for calculating RE^well‑mixed^ because it is the combination that yielded their lowest error results. As for comparisons with the work of Alvarado-Jiménez and Tasinato,^64^ we could only compare two compounds: HFC-134a and HFE-125. For these two cases, the error (RE^well‑mixed^ – RE^exp^) reported by Alvarado-Jiménez and Tasinato is 0.01 and 0.02 W m^–2^ ppbv^–1^, respectively. Rounding our RE results to two significant digits as done by Alvarado-Jiménez and Tasinato, we obtain errors of 0.01 and −0.02 W m^–2^ ppbv^–1^, respectively. While a sample size of only two molecules is insufficient for drawing definitive conclusions, a comparison of our results with those reported by Alvarado-Jiménez and Tasinato (using a highly robust and computationally demanding anharmonic approach), Bravo et al.,^57^ Betowski et al.,^60^ and Van Hoomissen et al.,^53^ suggests our method may offer an improved balance between accuracy and computational cost for estimating the radiative efficiency of structurally complex fluorinated molecules.

One aspect of the calculations that is also worthwhile commenting is the difference between an RE calculation considering only the lowest energy conformer and the RE calculation with the inclusion of all conformers. From the small set of 22 compounds with more than 1 conformer available, we observe a slight increase in the relative error [lowest conformer calculation with respect to the full conformer calculation of eq 17] as a function of the number of available conformers: 1.4% for compounds with six or less conformers and 3.0% for compounds with seven or more conformers. A more precise evaluation of this trend requires a much larger number of molecules, covering a vast number of available conformers.

### 3.2 Integrated Absorption Cross Sections

As mentioned above, although we do not fit the cross sections directly, our procedure does fit them indirectly via the *F*_*i*_(*ν̅*_*i*_) radiative forcing curve—which we can think of as acting as a weight function. It then becomes necessary to evaluate the difference between our M08-HX/pcseg-2 scaled calculations (λ^*F*^ = 0.978, *b* = 0.395 cm^–1^, λ^*I*^ = 0.855) for the cross sections and the unscaled calculations (λ^*F*^ = 1, *b* = 0 cm^–1^, λ^*I*^ = 1). We recall that we do not have the information of how the different cross-section data enters the calculation of the RE for each specific compound in the WMO2022 report. Because of this, and to make the proposed comparison the least problematic possible, we used the experimental integrated absorption cross sections for compounds that only had one source of data in the paper by Hodnebrog et al.^95^ We should also note that this experimental data was determined with varying values for *ν̅*_1_ and *ν̅*_2_ (see the Supporting Information of ref 95 where the values of *ν̅*_1_ and *ν̅*_2_ are given for each compound), while our integrated absorption cross-section values are calculated in the complete [0,3000] cm^–1^ range defined in eq 18. The results can be seen in Table 3.

**Table 3:** Comparison between the Experimental (Ref 95), Unscaled (*λ^F^* = 1, *b* = 0 cm^–1^, *λ^I^* = 1) and Scaled (*λ^F^* = 0.978, *b* = 0.395 cm^–1^, *λ^I^* = 0.855) M08-HX/pcseg-2 Integrated Absorption Cross Sections (*S*) for Selected Fluorinated Compounds from the CFM2025 Dataset

		*S* (10^–17^ cm^2^ molecule^–1^ cm^–1^)		
Formula	CASRN	Ref 95	(1, 0 cm^–1^, 1)	(0.978, 0.395 cm^–1^,0.855)
CH_2_FCH = CH_2_	818-92-8	4.40	5.05	4.32
CF_3_CH_2_OCF_3_	20193-67-3	33.5	38.5	32.9
CF_3_C(O)OCH = CH_2_	433-28-3	23.1	34.8	29.8
CF_3_C(O)OCH_2_CH_3_	383-63-1	25.4	29.1	24.8
CF_3_C(O)OCH_2_CH = CH_2_	383-67-5	23.7	30.5	26.1
CF_3_CHO	75-90-1	13.3	16.7	14.2
CF_3_CF_2_CHO	422-06-0	16.0	21.8	18.7
CF_3_CH_2_CH_2_CHO	406-87-1	17.0	21.4	18.3
CF_3_CF_2_CF_2_CHO	375-02-0	19.8	27.0	23.1
CF_3_C(O)CH_2_CH_3_	381-88-4	12.7	17.9	15.3
CH_3_C(O)CH_2_F	430-51-3	6.60	9.24	7.9
CF_3_C(O)CF_3_	684-16-2	24.6	28.0	23.9
CF_3_CF_2_C(O)CF(CF_3_)_2_	756-13-8	40.5	47.2	40.3

It is clear from the data presented in Table 3 that the scaled cross sections represent a significant improvement with respect to the unscaled ones. This improvement can be understood by looking at Figures 3 and 4, where we show, for ethyl 2,2,2-trifluoroacetate and 1,1,1,3,3,3-hexafluoropropan-2-one respectively, the differences between the experimental^100,101^ unscaled (λ^*F*^ = 1, *b* = 0 cm^–1^, λ^*I*^ = 1) and scaled (λ^*F*^ = 0.978, *b* = 0.395 cm^–1^, λ^*I*^ = 0.855) absorption cross-section spectra obtained in this work at the M08-HX/pcseg-2 level of theory. Each set of absorption cross-section spectra is superimposed to the radiative forcing^86^ per unit cross-section curve in an interval that highlights the overlap with the atmospheric window.

**Figure 3:**
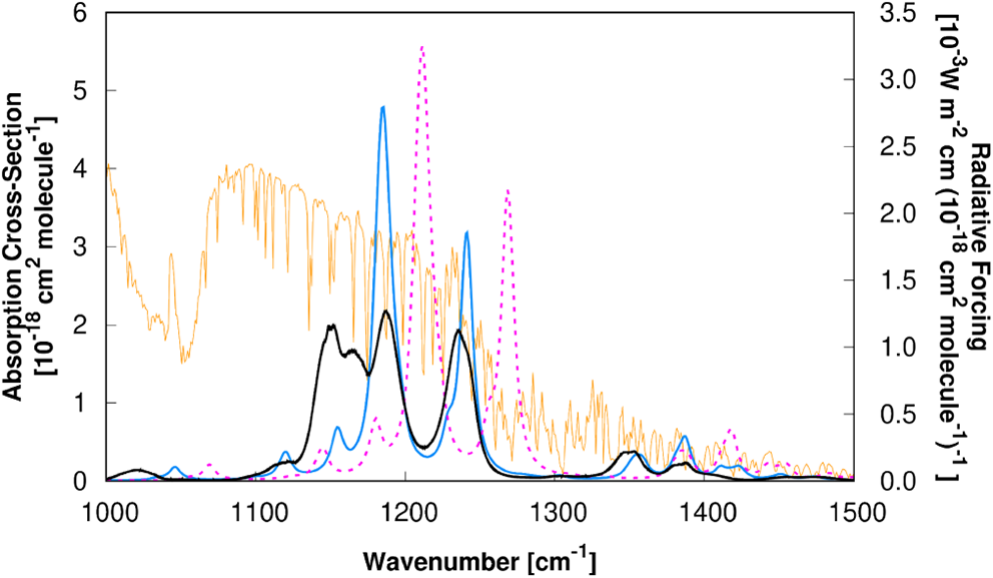
Comparison of experimental^100,101^ and theoretical cross sections for ethyl 2,2,2-trifluoroacetate. The experimental data (solid black line) are compared against theoretical results calculated in this work at the M08-HX/pcseg-2 level. The theoretical cross sections are presented in both unscaled (dashed purple line) and scaled (solid blue line) forms to facilitate a direct comparison of the effects of the scaling factors against the experimental results. The cross sections are overlaid with the radiative forcing^86^ per unit cross-section (solid orange line).

**Figure 4:**
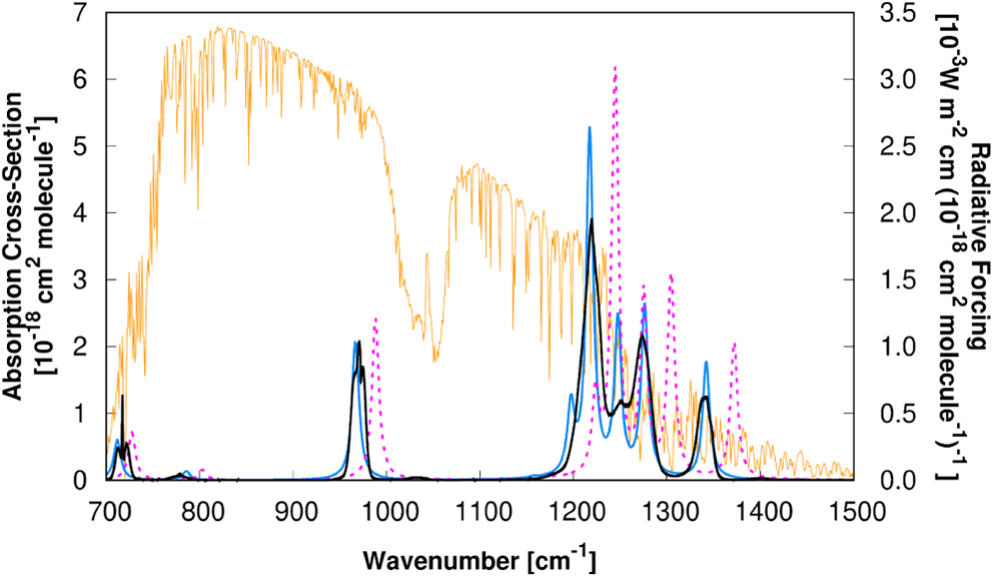
Comparison of experimental^100,101^ and theoretical cross sections for 1,1,1,3,3,3-hexafluoropropan-2-one. The experimental data (solid black line) are compared against theoretical results calculated in this work at the M08-HX/pcseg-2 level. The theoretical cross sections are presented in both unscaled (dashed purple line) and scaled (solid blue line) forms to facilitate a direct comparison of the effects of the scaling factors against the experimental results. The cross sections are overlaid with the radiative forcing^86^ per unit cross-section (solid orange line).

In both figures we can clearly see the effect of the scaling factors, which shift the unscaled cross sections to lower wavenumbers with a reduced intensity, clearly approaching the experimental cross sections. For ethyl 2,2,2-trifluoroacetate (Figure 3), the shift from the unscaled to the scaled cross-section leads to an improvement of the RE^well‑mixed^ value from 0.265 to 0.277 W m^–2^ ppbv^–1^, compared with the experimental value of 0.315 W m^–2^ ppbv^–1^. This difference to the experimental value is probably caused by the fact that our calculations cannot reproduce the experimental cross-section around 1150 cm^–1^. Here, anharmonic calculations are most likely the only way to effectively improve the RE^well‑mixed^ value. On the other hand, for 1,1,1,3,3,3-hexafluoropropan-2-one (Figure 4), the shift from the unscaled to the scaled cross-section leads to an improvement of the RE^well‑mixed^ value from 0.267 to 0.284 W m^–2^ ppbv^–1^, almost coinciding with the experimental value of 0.289 W m^–2^ ppbv^–1^. This excellent agreement can be rationalized from the huge overlap between the experimental and scaled cross sections in the atmospheric window.

### 3.3 Radiative Efficiencies beyond the CFM2025 Data Set

In order to test the λ^*F*^, *b* and λ^*I*^ parameters beyond the CFM2025 data set, we chose five additional fluorinated compounds of increased conformational complexity: an HFC of 17 atoms (HFC-43–10mee, CASRN of 138495-42-8), an hydrofluoroalcohol of 24 atoms (undecafluoroheptan-1-ol, CASRN of 185689-57-0), an hydrofluoroether of 16 atoms (HFE-338pcc13, CASRN of 188690-78-0), an hydrofluoroolefin of 18 atoms (3,3,4,4,5,5,6,6,6-nonafluorohex-1-ene, CASRN of 19430-93-4) and a perfluorocarbon of 23 atoms (PFC-61–16, CASRN of 335-57-9). The results are presented in Table 4. Notably, even for these larger molecules the errors remain low. For the five fluorinated compounds of Table 4 we obtain MAPE = 9.8% and MAE = 0.05 W m^–2^ ppbv^–1^. If we now consider these five molecules plus the CFM2025 data set we obtain MAPE = 6.4% and MAE = 0.02 W m^–2^ ppbv^–1^, just slightly above the errors for the CFM2025 data set alone, with its errors absorbing the errors associated with Table 4. Due to the quality of the results obtained with the parameters of Bravo et al.^57^ (see Table 2), we have also calculated RE^well‑mixed^ for these five extra compounds with their approach. It can be seen in Table 4 that our methodology yields a radiative efficiency closer to the experimental one for three cases, while the parameters of Bravo et al. yield a better result for CHF_2_OCF_2_CF_2_OCHF_2_. For the n-C_7_F_16_ molecule, both approaches yield the same result.

**Table 4:** Comparison between the RE^well‑mixed^ and GWP(100) Values Obtained for Five Extra Fluorinated Compounds beyond the CFM2025 Dataset: This Work, Obtained with *λ^F^* = 0.978, *b* = 0.395 cm^–1^ and *λ^I^* = 0.855, Ref 57 (*λ^F^* = 0.977, *b* = 11.664 cm^–1^ and *λ^I^* = 1.000) and the Most Recent Recommended Values Published in the WMO2022 report^26^

		RE (W m^–2^ ppbv^–1^)			GWP(100)	
Formula	# Conformers	Ref 26	This work	Ref 57	Ref 26	This work
CF_3_CHFCHFCF_2_CF_3_	8	0.369	0.373	0.382	1610	1531
CF_3_(CF_2_)_4_CH_2_CH_2_OH	204	0.371	0.458	0.470	<1	0.6
CHF_2_OCF_2_CF_2_OCHF_2_	114	0.904	0.768	0.910	3400	2586
n-C_4_F_9_CH = CH_2_	26	0.354	0.383	0.395	<1	0.2
n-C_7_F_16_	45	0.510	0.504	0.504	8610	7960

In addition to the compounds tested in Table 4, we were asked by one of the reviewers to extend our methodology to a few emerging greenhouse gases:^107^ heptafluoroisobutyronitrile [(CF_3_)_2_CFCN, CASRN of 42532-60-5], 1,1,1,2,2-pentafluoro-2-(trifluoromethoxy)­ethane (CF_3_OCF_2_CF_3_, CASRN of 665-16-7) and perfluoromethyl vinyl ether (CF_3_OCFCF_3_, CASRN of 1187-93-5). Trisna et al. report the following values: RE^well‑mixed^ = 0.201 ± 0.008 W m^–2^ ppbv^–1^ for (CF_3_)_2_CFCN, RE^well‑mixed^ = 0.544 ± 0.022 W m^–2^ ppbv^–1^ for CF_3_OCF_2_CF_3_ and RE^well‑mixed^ = 0.328 ± 0.013 W m^–2^ ppbv^–1^ for CF_3_OCFCF_3_. We report RE^well‑mixed^ values of 0.258, 0.450, and 0.326 W m^–2^ ppbv^–1^, respectively, which corresponds to relative errors of 28.3, 17.2 and 0.6%. A cautious interpretation of these results is essential, since our prior analyses utilizes the curated experimental RE^well‑mixed^ values from the WMO2022 report. The absence of this curation process in the current data set^107^ warrants caution when drawing conclusions based on these comparisons. We should add that a value for RE^well‑mixed^ of (CF_3_)_2_CFCN based on another experimental IR spectrum is reported in the WMO2022 document: 0.223 W m^–2^ ppbv^–1^. This lowers the error from 28.3 to 10.9%. We further investigated the 17.2% error for CF_3_OCF_2_CF_3_, as Trisna et al. were the first to report the RE^well‑mixed^ value for this compound in their very recent paper.^107^ Figure 5 shows the comparison between the experimental and theoretical IR spectra.

**Figure 5:**
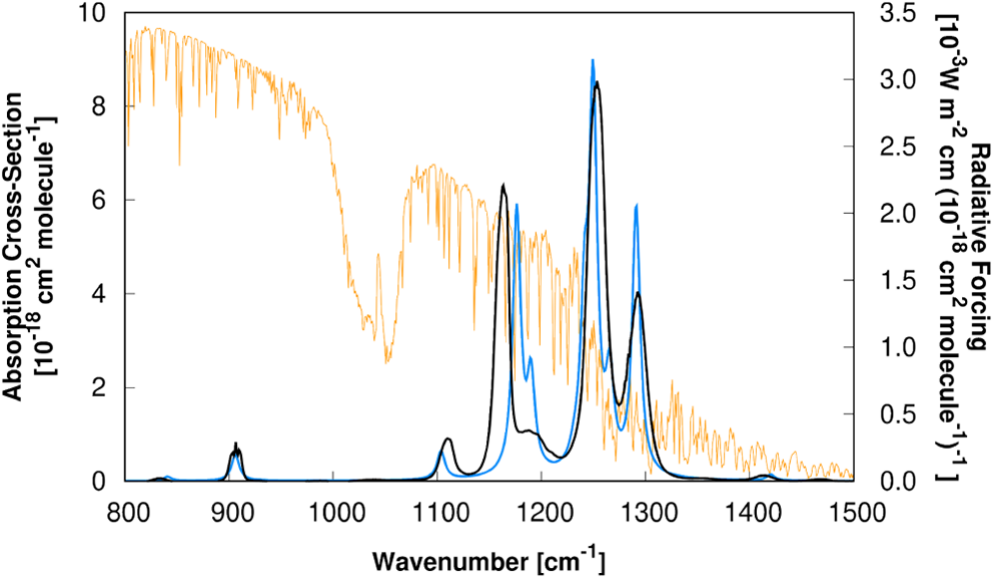
Comparison of experimental^107^ and theoretical cross sections for CF_3_OCF_2_CF_3_. The experimental data (solid black line) are compared against theoretical (scaled) results calculated in this work at the M08-HX/pcseg-2 level (solid blue line). The cross sections are overlaid with the radiative forcing^86^ per unit cross-section (solid orange line).

Both spectra show a good agreement except, interestingly, in the region between 1100 and 1200 cm^–1^, similar to what we observed for ethyl 2,2,2-trifluoroacetate (Figure 3). This small difference between both spectra is enough the cause the 17.2% error in the value of RE^well‑mixed^. A more definitive assessment will be possible only once the updated benchmarks from the forthcoming World Meteorological Organization report become available.

### 3.4 GWP Results Based on MC-TST/CTSR Lifetimes and Respective Uncertainties

Finally, in this subsection we present the results concerning the calculation of GWP_*X*_(100) (henceforth simply referred to as GWP_*X*_ in the equations below) for three compounds of the CFM2025 data set: the HFE-236ea2 ether CHF_2_OCHFCF_3_ and two alcohols, CF_3_CF_2_CH_2_OH and CHF_2_CF_2_CH_2_OH. However, in this case, the atmospheric lifetimes were determined by resorting to the respective previously published^42^ MC-TST/CTSR OH oxidation rate constants, *k*_OH_(calc). We extracted the respective Arrhenius-Kooij parameters from Table 1 of ref 42 and calculated the three rate constants at 272 K.^12,108^ The lifetimes were then obtained via eq 4, with the associated Δτ_*X*_ error being given by

22 $$\Delta \tau_{X}=\sqrt{{[\frac{\partial \tau_{X}}{\partial k_{\mathrm{OH}}\left(\mathrm{calc}\right)}]}^{2}\Delta k_{\mathrm{OH}}^{2}\left(\mathrm{calc}\right)}=\sqrt{\frac{\Delta k_{\mathrm{OH}}^{2}\left(\mathrm{calc}\right)}{{[\mathrm{OH}]}^{2}k_{\mathrm{OH}}^{4}\left(\mathrm{calc}\right)}}$$

where we have ignored the error associated with [OH]. Here, Δ*k*_OH_(calc) is the error associated with each rate constant, calculated with eq (30) of ref 42 for an updated σ_*B*_ = 138 K value, which reflects the MAE^109^ concerning the lowest hydrogen-abstraction barrier heights for the set of reactions studied so far^42,43^ with the MC-TST/CTSR protocol.

The uncertainty in the GWP_*X*_ value is given by

23 $$\frac{\Delta {\mathrm{GWP}}_{X}}{{\mathrm{GWP}}_{X}}=\sqrt{{\left(\frac{\Delta {\mathrm{AGWP}}_{X}}{{\mathrm{AGWP}}_{X}}\right)}^{2}+{\left(\frac{\Delta {\mathrm{AGWP}}_{{\mathrm{CO}}_{2}}}{{\mathrm{AGWP}}_{{\mathrm{CO}}_{2}}}\right)}^{2}}$$

with the uncertainty of ${\mathrm{AGWP}}_{{\mathrm{CO}}_{2}}\left(100\right)$ (second term in the square root) having an estimated value^25^ of 26%. The calculation of ΔAGWP_*X*_ is expressed as^25^

24 $$\Delta {\mathrm{AGWP}}_{X}=\sqrt{{\left(\frac{\partial {\mathrm{AGWP}}_{X}}{\partial {\mathrm{RE}}_{X}^{\mathrm{well‐mixed}}}\Delta {\mathrm{RE}}_{X}^{\mathrm{well‐mixed}}\right)}^{2}+{\left(\frac{\partial {\mathrm{AGWP}}_{X}}{\partial \tau_{X}}\Delta \tau_{X}\right)}^{2}}$$

with several factors affecting the ${\mathrm{RE}}_{X}^{\mathrm{well‐mixed}}$ and τ_*X*_ uncertainties. Contributions to the ${\mathrm{RE}}_{X}^{\mathrm{well‐mixed}}$ uncertainties (Table 1 of ref 95) can be associated with experimental absorption cross sections (neglected far IR and shortwave bands), radiation scheme, clouds, spectral overlap and water vapor distribution, surface emissivity and temperature, atmospheric temperature, tropopause level, temporal and spatial averaging, stratospheric temperature adjustment and nonuniform vertical profile. Contributions to the τ_*X*_ uncertainties can generically be attributed^110^ to the uncertainty associated with OH concentration, reaction rate coefficients (namely the extrapolation of the laboratory based determinations to low atmospheric temperatures), stratospheric photochemical loss determinations, stratospheric transport and circulation, and radiative transfer and atmospheric opacity. In what follows we will focus on the theoretical contributions to the uncertainties.

The partial derivatives of eq 24 were calculated numerically with the central differences formula. For the ΔRE_*X*_ error we used^109^ our calculated MAE value of 0.014 W m^–2^ ppbv^–1^. The results are given in Table 5.

**Table 5:** Comparison between the GWP(100) Values of Three Fluorinated Compounds from the CFM2025 Dataset as Reported in WMO2022 ^26^ and as Calculated in This Work but Incorporating the Lifetimes Obtained with Our MC-TST/CTSR protocol^42^ ^a^

	τ		GWP(100)		ΔGWP(100)
Formula	Ref 26	Ref 42	Ref 26	This work	This work
CF_3_CF_2_CH_2_OH	168 days	216 days	35	45	26
CHF_2_CF_2_CH_2_OH	92.4 days	36 days	16	3	2
CHF_2_OCHFCF_3_	13.7 years	21.5 years	2530	3354	1848

a In addition we also include the ΔGWP(100) error estimation.

For each molecule, an analysis of the two terms present in the square root of eq 24 shows that the largest contributions for ΔGWP come from the term that depends on Δτ. The difficulties in theoretically calculating the atmospheric lifetimes are vastly recognized in the literature, with the discussion about the uncertainties in the experimental values of GWP being also common.^25,26,95,111^ However, a discussion and presentation of errors for theoretically calculated lifetimes and radiative efficiencies and how they propagate in the respective GWP calculations is rarely (if ever) seen in theoretical and computational investigations. Although our analysis was limited to three compounds, the results are encouraging as the predicted GWP values are generally in qualitative agreement with the experimental values, especially considering the large uncertainties inherent to the atmospheric lifetimes. We add that the rate constant *J* for photolysis would be necessary for lifetime calculation in fluorinated ketones and aldehydes.

In this article, we have developed a methodology for performing low error calculations for the radiative efficiency of several different classes of fluorinated molecules. Our approach uses the most recent radiative forcing curve and can be applied to any combination of electronic structure method and basis set (model chemistry). Our method uses all available conformer population at 298 K for each compound, thus approximately replicating the existent fractional population of molecules when the experimental IR spectra are obtained. By minimizing the relative error of the radiative efficiency for a data set of 38 fluorinated compounds of different classes, we can easily find optimum values for the vibrational frequency and intensity scaling parameters for a given model chemistry without the need to go beyond the harmonic approximation, keeping the procedure computationally feasible. The application of this procedure to M08-HX/pcseg-2 calculations allows us to effortlessly incorporate accurate radiative efficiency calculations to our MC-TST/CTSR protocol and thus obtain theoretical values of the GWP metric. These GWP results are given with error bars, which together with the quality of the atmospheric lifetimes and radiative efficiencies can be seen as an upgrade to the current theoretical methodologies that calculate GWP values.

## 4 Implications and Future Perspectives

The methodology developed in this study enables a theoretical estimation of the radiative efficiency for the diverse classes of fluorinated compounds relevant to the Kigali Amendment to the Montreal Protocol with an accuracy that, to the best of our knowledge, surpasses existing approaches based on the harmonic approximation. The high reliability of this method supports its application in scenarios where experimental IR spectra are either inaccessible or lack the necessary accuracy. Furthermore, the designed protocol for the calculation of the scaling parameters in Section 2.3 enables straightforward recalibration of the computed scaling factors, should the experimental radiative efficiency values or the radiative forcing curve undergo what is deemed a substantial revision by the scientific community. This adaptability enhances the method’s long-term applicability and the use of three scaling parameters may offer advantages over the more rigid approaches that employ just two.

While the quantified uncertainties for the GWP metric are substantial for direct use in regulatory compliance (e.g., strict application of thresholds mandated by the EU’s F-gas regulation (EU) 2024/573), our methodology provides crucial early stage screening and prioritization, rapidly identifying high-risk and promising candidates to focus and accelerate subsequent resource-intensive experimental measurement. This is particularly relevant for the phasedown of HFCs, as mandated by the Kigali Amendment, where reliable theoretical predictions and their uncertainty estimates can complement experimental data to efficiently identify environmentally safer alternatives from thousands of potential compounds. Ultimately, our approach contributes to a more precise evaluation of the radiative effects of fluorinated compounds, aiding global efforts to mitigate climate change.

## Supplementary Material



## Notes

Cartesian coordinates for all calculated rotamers, with each coordinate file also containing the M08-HX/pcseg-2 electronic energy, the Gibbs correction, and the corresponding integrated absorption cross-section; the respective IR cross-section data for each rotamer; the final Boltzmann weighted IR cross-section; a results file containing the relative Gibbs energies for all rotamers; the integrated absorption cross-section corresponding to the final Boltzmann weighted IR cross-section; the well-mixed radiative efficiency values (contribution of all conformers and of the lowest energy one); global warming potential values and their derivatives (ZIP)
