# Supplementary material for: Modeling Radiative Efficiency across Fluorinated Molecules: Bridging Chemistry and Climate Policy for Global Warming Potential Estimations
Source: Environ Sci Technol. 2026 Feb 16;60(8):6188–202. doi: 10.1021/acs.est.5c16501 (PMC12961741; doi:10.1021/acs.est.5c16501)
Supplement: Supplementary file 1 [file es5c16501_si_001.zip › Supporting.Information/Supporting.Information.pdf]

Supporting Information: Modeling Radiative Efficiency Across Fluorinated Molecules: Bridging Chemistry and Climate Policy for Global Warming Potential Estimations

Luís P. Viegas* and Matilde A. Susano

Coimbra Chemistry Centre-Institute of Molecular Sciences (CQC-IMS), Department of Chemistry, University of Coimbra, 3004-535 Coimbra, Portugal

E-mail: lpviegas@ci.uc.pt

This Supporting Information contains the data concerning all compounds for which we calculated the radiative efficiency. For each compound there is a specific folder with the following information:

- Cartesian coordinates for all calculated rotamers, with each rotamer_i.xyz file (where i is the index) containing, besides the coordinates (in Angstrom), the M08-HX/pcseg-2 electronic energy, the Gibbs correction and the corresponding integrated absorption cross-section

- The respetive infrared cross-sections for each rotamer in cross-section_i.dat [wavenumber (cm^{-1}) vs cross-section (cm^2 $molecule^{-1}$)]

- The final Boltzmann weighted infrared cross-section in the file wspectra.dat [wavenumber (cm^{-1}) vs cross-section (cm^2 $molecule^{-1}$)]

- A results file (RESULTS.txt) containing the relative Gibbs energies for all conformers, the integrated absorption cross-section corresponding to the final Boltzmann weighted infrared cross-section, the well-mixed radiative efficiency values (contribution of all conformers and of the lowest energy one), global warming potencial values and their derivatives
